# High Densification of Tungsten via Hot Pressing at 1300 °C in Carbon Presence

**DOI:** 10.3390/ma15103641

**Published:** 2022-05-19

**Authors:** Oleksii Popov, Vladimir Vishnyakov

**Affiliations:** 1 Metal Physics Department, Faculty of Physics, Taras Shevchenko National University of Kyiv, 01033 Kyiv, Ukraine; 2 SRC “Synthesis”, 02161 Kyiv, Ukraine; 3 Institute for Materials Research, University of Huddersfield, Huddersfield HD1 3DH, UK; v.vishnyakov@hud.ac.uk

**Keywords:** tungsten, reactive hot pressing, powder milling, densification kinetics, tungsten hemicarbide, plasma-facing material

## Abstract

A reactive sintering technique with a small addition of carbon (up to 1.9 wt.%) has been used for tungsten powder consolidation. The process allowed procurement of the nonporous and fully densified material at 1300 °C and 30 MPa in 12 min. The SEM and EDX analysis showed that the milling of 5 μm tungsten powder with 0.6, 1.3, and 1.9 wt.% of carbon in a planetary mill led to the formation of the nanostructured mix, which appears to be W-C nanopowder surrounding tungsten grains. X-Ray Diffractometry data indicated tungsten hemicarbide (W_2_C) nucleation during the hot pressing of the milled powders. The exothermic reaction 2W + C → W_2_C occurs during the sintering process and promotes charge densification. The Vickers hardness and indentation toughness of W-1.3 wt.%C composition reached 5.7 GPa and 12.6 MPa∙m^1/2^, respectively. High toughness and high material densification allow proposing the W-WC_2_ for use as a plasma-facing material in fusion applications.

## 1. Introduction

Erosion of the plasma-facing material is regarded as one of the dominant lifetime issues in a fusion reactor [1]. In many respects, tungsten is one of the best potential candidates for first-wall applications and plasma-facing surfaces. Tungsten produces a relatively low amount of plasma impurities and has a low ion sputtering yield [2]. The crucial issue hampering tungsten use is its brittleness [3]. The brittleness is further amplified after high-temperature annealing by the grain growth. On the other hand, poor sinterability contributes to the product’s high cost, motivating a search for ways of tungsten manufacturing simplification.

There are several tungsten alloys which allow sintering temperature reduction from 2500 °C for pure tungsten [4] to as low as 1300 °C for 96% density of a W-Ni-Fe-Co mechanically alloyed system [5], 1200 °C for 98% density of a W-Ni-Cu-Mn system [6], and even 1100 °C for 96% density of a W-Cu-Sn-Fe system [6]. However, most reduced sintering temperature alloys contain more than 10 wt.% of low-melt additives introduced into the material matrix. The additives affect not only the composite high-temperature stability but also hamper the alloys’ radiation endurance [7] and thus can hardly be a feasible proposition for the plasma-facing material.

The most studied way of almost pure high-density tungsten sintering temperature decrease is the use of additives, which remain segregated on the W–W powder grain interfaces and in which tungsten has some substantial solubility. Such additives, with the content of approximately 1 wt.%, act as short-circuit diffusion pathways, thereby promoting densification [6], and present so-called chemical sintering activators. Li and German [8] produced W-0.3 wt.%Ni with a density of 96% after 1 h of sintering at 1200 °C. However, the authors showed that the main problem of the chemically activated sintering is rapid structure coarsening close to full density. This highlights the fact that the sintering aids targeting tungsten diffusion and provides additional favourable conditions for the future main phase recrystallization with additional detrimental mechanical properties. Indeed, an essential strength reduction of the recrystallized material was presented in [8].

Another traditional way of powder densification improvement is the application of external pressure. Majumdar et al. [9] managed to densify pure tungsten micro powder after hot pressing at 1950 °C and 120 MPa for 2 h, significantly decreasing the sintering temperature as compared to Braun and Sedlatschek’s [4]. It is still considered that further sintering parameter reduction remains a very important problem [10].

Mondal et al. [11] reported densification of pure tungsten nanopowder to 95% density at 1600 °C for 30 min with the use of microwave activated sintering. The grains grew from 70 nm to 2.6 μm during the process. A field-assisted sintering technique applied to pure 0.6–0.9 μm tungsten powder allowed obtaining material with 98.5% density and 22 μm grains at 2000 °C, with a pressure of 85 MPa for 30 min [12]. At the same time, the addition of 10 vol.% of WC resulted in 99% density and 3μm grains at 1700 °C, with a pressure of 85 MPa for 5 min. Further temperature and time lowering were achieved within the SPS process by Senthilnathan et al. [13], leading to W+1%Ni being consolidated to 90% density at 1200 °C and 30 MPa for 3 min. The material, however, contained Ni, which was shown to accelerate the tungsten recrystallization [8].

It is possible to see that several sintering promotion methods (i.e., applied pressure, sintering aids, electromagnetic field, etc.) can be used together to provide greater results than any single one. In addition, for reasonable plasma-facing material performance, the sintering aids should not contain phases resulting in better tungsten atom diffusion and grain coarsening.

To date, there are few, if any, attempts to investigate the influence of in-situ chemical reactions on the densification kinetics of tungsten alloys while, as shown in other cases [14,15,16], reactive sintering presents some extremely effective mechanisms of consolidation improvement.

Within the presented work, we demonstrate the potential of hot pressing assisted by the in situ chemical reaction for the creation of W-based plasma-facing materials. We chose the W-C system as tungsten carbide is known to be an efficient sintering aid [12,17] and does not have any evident negative impact on tungsten high-temperature characteristics, and tungsten carbide is reasonably stable under ion irradiation [7]. The WC formation from the elemental powders is an exothermic process, the reaction energy of which is known to be important for the reactive sintering consolidation kinetics [16].

## 2. Materials and Methods

Commercially available powders of W (grain size at ~5 μm) and carbon black produced by Sigma-Aldrich, UK, were used as starting materials. The material purity was around 99.9%. The starting powders (e.g., green bodies) of 4 different compositions (see Table 1) were milled in a planetary mill (WC jar, WC balls, ball to powder weight ratio 10:1) at 500 rpm for 6 h.

The sintered samples were produced by green body hot-pressing at temperatures of 900 °C, 1300 °C, and 1850 °C. The sintering pressure in the graphite die was 30 MPa. The die assembly was heated with AC (50 Hz) current in a vacuum with the use of the hot-pressing equipment DCS-1 produced by SRC Synthesis (Kyiv, Ukraine) and the Institute for Materials Research (Huddersfield, UK). The heating rate (after preheating to 300 °C for 20 min) was approximately 200°/min. The isothermal dwelling time for the different samples is presented in Table 1. The hot-pressed specimens were in the form of discs of 10 mm diameter and 4 mm height.

The crystalline phases were identified by X-Ray Diffraction (XRD). The sintered ceramic bulk densities were measured using the Archimedes method and the theoretical densities (ρth) were estimated according to the rule of mixtures based on the XRD results for the phase composition. Analysis of the microstructure was performed by Scanning Electron Microscopy (SEM) of the sample fracture surfaces. Vickers hardness measurements were performed with a load of 9.8 N for 15 s on polished surfaces. The fracture toughness was estimated by measuring the crack lengths generated by the Vickers indentations with a load of 147 N. The toughness was calculated according to the formula of Evans and Charles [18]. The heat effect and adiabatic temperature were calculated using thermochemistry data from the NIST Chemistry WebBook [19].

## 3. Results and Discussion

### 3.1. Initial Powder Evolution during Milling

**Figure 1:**
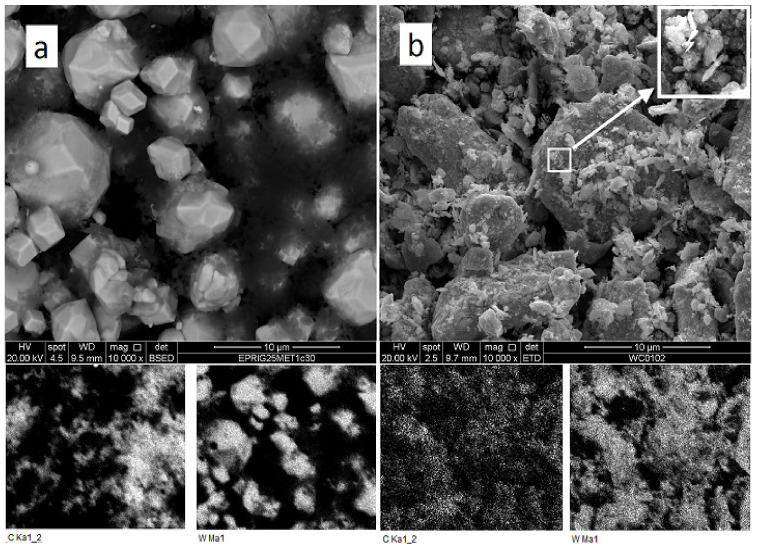
SEM and EDX analysis of as-mixed (**a**) and milled (**b**) W + 1.3% C powder.

**Figure 2:**
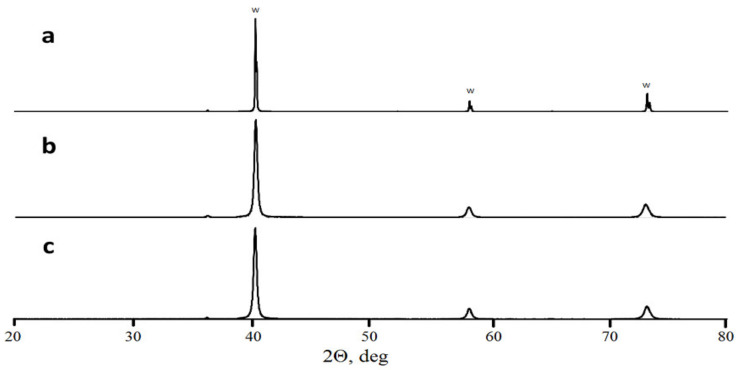
X-ray diffractometry of as-mixed W powder (**a**), milled W powder (**b**), and milled W + 1.3% C powder (**c**).

A comparison of as-mixed and milled W + 1.3% C powders (Figure 1) shows an essential change in carbon and tungsten distribution after the milling. In as-mixed powder, carbon-rich areas on the EDX-maps can be seen separately from the tungsten-rich ones. Carbon peak was not seen on the XRD pattern of the powder, either due to carbon being amorphous and/or because of the too small amount of carbon mixed with tungsten. At the same time, carbon and tungsten maps of the milled powder entirely overlap. The tungsten grain appearance also changes, in essence, from seen 3–10 μm monocrystals to shapeless particles with sizes between 0.1 and 10 μm. As shown in [20,21], the disappearance of carbon as a separate phase during the milling procedure can be explained by the WxC nanopowder formation. This would not be surprising because mechanical alloying is a well-known process. Given that the material will be in the predominantly amorphous state, it is no surprise that the XRD spectra of milled powder show neither any hints of new crystalline phase formation nor C in W solid solution peak position shift (Figure 2). At the same time, the milled powder XRD peaks are broadened as compared to the XRD peaks from as-mixed powders. The broadening can be caused by either micro-stress accumulation or considerable grain size refinement.

**Figure 3:**
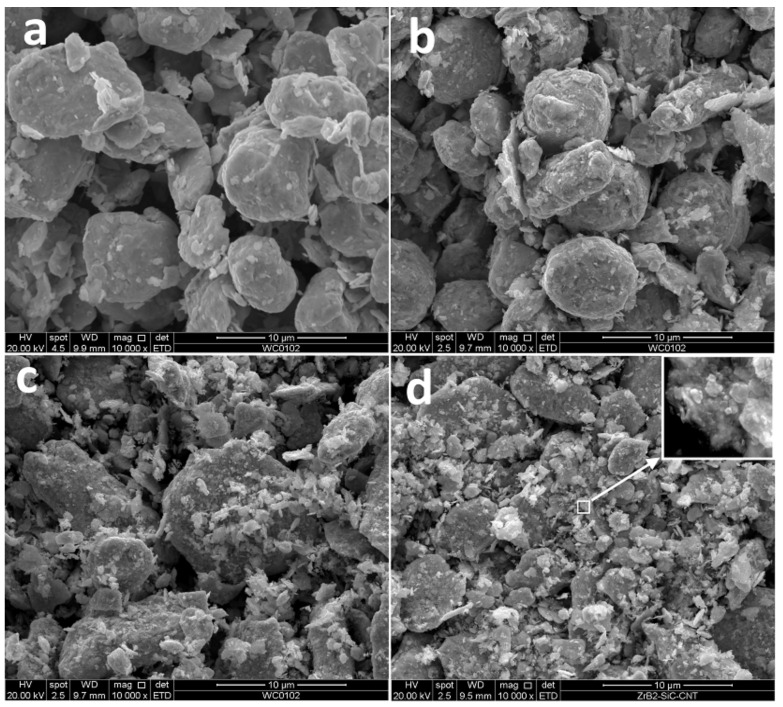
Secondary electron images of milled powders: (**a**) pure tungsten, (**b**) W + 0.6% C, (**c**) W + 1.3% C, (**d**) W + 1.9% C.

The milled tungsten powder (Figure 3a) consists of grains with an average size close to 5 μm. The SEM images demonstrate that the milling procedure did not lead to the powder grain size refinement; however, the plastic deformation visibly changed the grain shapes. The milling of W-C powders led to the formation of submicron particles, the amount of which increased with carbon content. The exact average grain size is difficult to calculate. One can only, with certainty, say that the grain surface is nanostructured (see Figure 3d). Earlier, He et al. [22] reported the existence of clear XRD peaks from 5 nm tungsten carbide crystals. Similar peaks are not observed in this work, and we have to assume that the presented nanoparticles can hardly be tungsten carbide nuclei (see Figure 2). As shown by Kurlov and Gusev [23], carbon in crystalline tungsten equilibrium solubility does not exceed 0.1%. The only sensible explanation for carbon phase absence on the milled powder SEM images (Figure 1b) is W-C extra-fine mixture with carbon particles less than several nanometres. Considering the pure tungsten milled powder structure (Figure 3a), we can hardly expect the mixture formation inside the bulk particles. Hence, after milling, powders 3–7 consist of W-based grains with nanostructured W-C mix covering. It should be stressed that if the formed carbide phase is amorphous then it will be close to impossible to identify the phase by XRD in the mixture and presence of strong crystalline peaks. TEM analysis is planned to investigate the milling processes further.

### 3.2. The Charge Consolidation and Sintered Materials Structure

**Figure 4:**
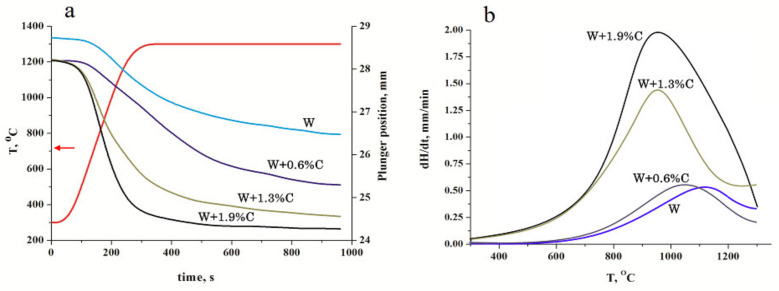
Sample heights (**a**) and the charge densification speed (**b**) vs. sintering time and temperature for tungsten powder with different content of carbon.

The dependence of the charge shrinkage on sintering time for the samples pressed at 1300 °C (see Figure 4) shows that carbon addition improves the densification kinetics significantly. Considering that the green body amounts were weighted to obtain 4 mm nonporous samples, carbon presence increased even the green body density. At temperature rise, the undoped tungsten consolidation started at approximately 1000 °C, which corresponds to the temperature of the tungsten yield strength reduction [24] and occurs by the plastic deformation of the most stressed grain-contact areas. As shown in [24], the yield strength at 1300 °C does not exceed 30% of that of the room temperature and the overall charge shrinkage approximates 2.3 mm, resulting in a sample density of 13.6 g/cm^3^ (see Table 1). An addition of 0.6% carbon did not increase the densification speed but extended the period of intense consolidation and led at last to shrinkage of 2.8mm and a material density of 16.76 g/cm^3^. Further carbon addition led to an essential rise of the densification speed up to 1.5 and 2 mm/min (Figure 4b) and the overall shrinkage of 3.6 and 3.8 mm, respectively. It should also be noted that the maximum acceleration of the consolidation process shifted to lower temperatures and can be observed at approximately 900 °C. As shown in [25], such consolidation behaviour can be attributed to the in situ reaction.

**Figure 5:**
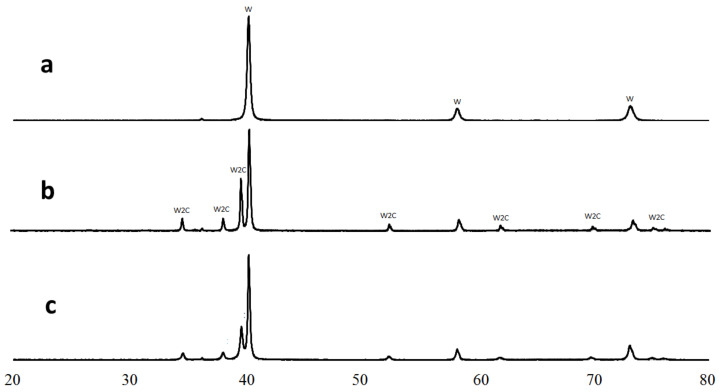
X-ray diffractometry of W + 1.3%C composition: milled powder (**a**), sample 5 sintered at 900 °C (**b**), and sample 4 sintered at 1300 °C (**c**).

As shown in [23], at temperatures lower than 1250 °C, the only thermodynamically stable W-C phase is tungsten carbide WC. However, the XRD-analysis of the sintered samples (see Figure 5c) indicated tungsten hemicarbide W2C existence alongside tungsten. Moreover, the sample produced at 900 °C also contained only the W2C phase with no hints of thermodynamically more stable WC crystals (see Figure 5b).

The similar results obtained by Fei et al. [26] were explained by carbon in tungsten accumulation proceeding inevitably via the atomic ratio of W:C = 2:1. The W2C phase has lower Gibbs energy than C in W solid solution, which leads to the following hemicarbide forming reaction:

2W + C → W_2_C (1)

in the case when an appropriate C concentration is present in the green body. The diffusivity of carbon in W2C was shown to be an order of magnitude less than that of carbon in pure tungsten [27,28]. Therefore, as soon as the hemicarbide is formed, further carbon accumulation in hemicarbide slows down, and this prevents WC nucleation. The reaction (1) heat effect is ΔH ≈ −30 kJ/moll [29]. Based on the W2C heat capacity presented in [30], the calculated reaction (1) adiabatic temperature is at around 350 °C. The reaction-induced temperature rise, ΔTr (see Table 2), does not exceed 250 °C and, in itself, can hardly result in considerable consolidation improvement.

As shown above, the nanostructured W-C mix in the milled initial powders surrounds the W particles. The reaction (1) dilatometry is approximate −11% (the density of nonporous composite based on the initial 2W + C mixture is 10.9% lower than that of W2C). The reaction-induced volume reduction on grain boundaries in the presence of external pressure promotes charge mobility and intensifies densification. Sample 6, including only 27 mol.% of the reactively formed (W2C) phase, has 10% porosity, while samples 4 and 7, with 50 and 69% of W2C, respectively, achieved the full density at 1300 °C (see Table 2).

### 3.3. The Sintered Materials Structure and Mechanical Characteristics

**Figure 6:**
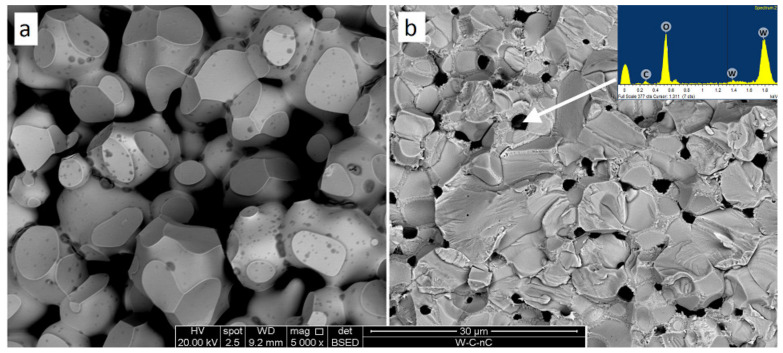
Fracture surfaces of pure tungsten (**a**) and W + 1.3% C with the point EDX analysis (**b**). Both samples (Samples 1 and 3) were sintered at 1850 °C. BSE images.

The structure of tungsten micro-powder hot-pressed at 1850 °C (Figure 6a) consists of ~15 μm tungsten grains with ~1 μm darker (supposedly oxide) spots. A considerable amount of open porosity corresponds to the sample density (see Table 2), showing the charge to be at the second stage of the consolidation process. The inter-crystallite cleavage illustrates poor adhesion of the sintered particles. In contradiction, sample 3 (W-1.3% C, 1850 °C, Table 1, Figure 6b) fracture surface presents a nonporous solid body with light-grey tungsten-based grains and dark ~3 μm oxide inclusions. The particle adhesion is strong enough to facilitate trans-crystallite cleavage.

**Figure 7:**
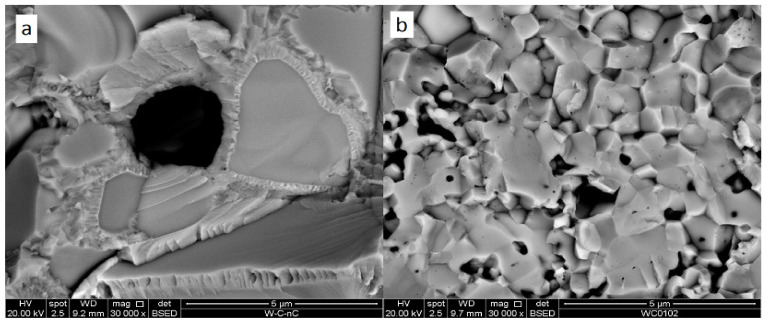
Fracture surfaces of W + 1.3% C materials sintered at 1850 °C (**a**) and 1300 °C (**b**) (Samples #1 and #4 correspondingly).

At a higher magnification, the structure of sample 3 demonstrates monocrystalline areas of 2–5 μm surrounded by supposedly polycrystalline covering (Figure 7). Considering the initial powder images (see Figure 3), the monocrystals can be pure tungsten. The surrounding polycrystalline is then W_2_C phase. Sample 4 containing the same composition sintered at 1300 °C demonstrates a significantly finer structure with submicron black oxide inclusions and 0.3–2 μm W and W_2_C grains.

Microhardness and fracture toughness of the sintered compositions estimated with the rule of mixture basing on pure tungsten (Hv = 4.2 GPa [11]; K1C = 5.1 MPa∙m^1/2^ [3]) and tungsten hemicarbide (Hv = 17 GPa; K1C = 3.6 MPa∙m^1/2^ [31]) presented in Table 2 substantially differed from the experimental results. The low parameters of samples 1 and 6 should be based on their porosity. However, composites 3, 4, and 7 have complete density. The microhardness values of all the mentioned materials are approximately half of the theoretical ones. This means that the dislocations are mobile at lower stresses, resulting in higher material plasticity. Better material plasticity would increase the plastic zone near the crack tip, improving the material fracture resistance. The indentation toughness values being significantly higher than the theoretically estimated ones confirm the later statement. The reactively obtained composite structure efficiently resists crack propagation.

## 4. Conclusions

Hot pressing of tungsten with the small addition of carbon (up to 1.9 wt.%) has been used for tungsten powder consolidation. Milling 5μm tungsten powder with 0.6, 1.3, and 1.9 wt.% of carbon in a planetary mill allows for creating a specific green body with a possible WxC nanostructured mixture surrounding tungsten grains. Sintering via the hot pressing route at 1300 °C and 30 MPa is assisted by tungsten–carbon reaction, which allows obtaining completely densified material within 12 min. XRD indicated tungsten hemicarbide (W2C) nucleation during the hot pressing of the milled powders. SEM imaging shows that nanostructured hemicarbide forms connecting tissue around tungsten grains. Reduced, as compared to theoretical, the material hardness of 5.7 GPa allows for high plasticity. This in turn increases resistance to crack development and increases composite toughens. The fully dense material with high toughness suggest that the W-WC_2_ can be used as a plasma-facing material in fusion applications.

## Figures and Tables

**Table 1:** The composition, sintering parameters, and density of the samples.

	Initial Powder Composition, wt.%		Sintering Parameters			ρ, g/cm^3^
№	W	C	T, °C	P, MPa	t, min	
1	100	0	1850	30	2	15.2
2	100	0	1300	30	12	13.6
3	98.7	1.3	1850	30	2	17.98
4	98.7	1.3	1300	30	12	17.97
5	98.7	1.3	900	30	12	10.8
6	99.4	0.6	1300	30	12	16.76
7	98.1	1.9	1300	30	12	17.63

**Table 2:** The material’s final composition, density, adiabatic temperature rise (ΔT_r_), and measured mechanical characteristics (H_v_, K_1C_). The theoretical values of Vickers hardness (H_vth_) and toughness (K_1Cth_) were estimated with the rule of mixture.

Sample	Composition, mol.%		ρ, g/cm^3^	ρ/ρ_th_, %	ΔT_r_, °C	H_v_, GPa	H_vth_, GPa	K_1C_, MPa∙m^1/2^	K_1Cth_, MPa∙m^1/2^
	W	W_2_C							
1	100	0	15.2	78.9	0	1.4	4.2	-	5.1
2	100	0	13.6	70.5	0	-	-	-	-
3	50	50	17.98	100	178	6.3	12	12.5	4.2
4	50	50	17.97	100	178	5.7	12	12.6	4.2
5	50	50	10.8	60	178	-	-	-	-
6	73	27	16.76	90.5	97	4.4	8.9	8.3	4.5
7	31	69	17.63	100	245	8.7	14.1	10.6	3.9

## Data Availability

The data presented in this study are available on request from the corresponding author.
